# A Review of Exercise Interventions for Rehabilitation in Drug‐Dependent Individuals

**DOI:** 10.1111/adb.70098

**Published:** 2025-12-10

**Authors:** Qinghua He, Li Zhu, Zhaosong Wang, Hao Wang, Yiyi Jiang, Xin Wang, Fuxuan Luo, Chaoyi Zhu, Changlong Zhan

**Affiliations:** ^1^ Guangxi Minzu University Nanning China; ^2^ Guangxi College of Sports Education Nanning China; ^3^ The Sixth Compulsory Isolation and Rehabilitation Center of Guangxi Nanning China

**Keywords:** benefit mechanisms, drug addiction, exercise intervention, exercise‐based drug rehabilitation

## Abstract

This study systematically investigates the multidimensional rehabilitative effects and neurobiological mechanisms of exercise interventions in individuals with substance use disorders (SUDs). By synthesizing and critically analysing current evidence, the aim is to establish a theoretical framework for exercise‐based rehabilitation and provide empirical support for optimizing intervention strategies. A comprehensive literature review was conducted, encompassing 39 core studies on exercise interventions in drug rehabilitation. Evidence‐based medicine principles were applied to integrate mechanistic findings and evaluate effect sizes. The review focused on examining the physiological, psychological and neurobiological effects of various exercise modalities in individuals with SUD. Findings consistently demonstrate that exercise interventions are effective in reducing drug craving and withdrawal symptoms, improving overall quality of life and potentially lowering relapse rates. For individuals undergoing rehabilitation, exercise promotes improvements in physical health, psychological well‐being and social functioning, while concurrently attenuating relapse vulnerability. As a nonpharmacological, multitarget intervention, exercise therapy exhibits significant potential in promoting neuroplasticity and facilitating psychological recovery in individuals with SUD. Standardized exercise prescriptions should be integrated into existing rehabilitation frameworks. Future research should emphasize comparative effectiveness across exercise modalities, explore the benefits of multimodal interventions, and further elucidate the interplay between biological, clinical and psychosocial mechanisms to enhance long‐term rehabilitation outcomes.

## Graphical abstract

The analysis of 39 core studies indicates that diverse exercise interventions have varying positive effects across different dimensions for various addiction populations. Exercise can exert beneficial impacts on the neurobiological mechanisms, as well as physiological and psychological aspects, of individuals with drug addiction. Different exercise modalities offer distinct advantages, and multi‐modal interventions demonstrate more comprehensive efficacy.

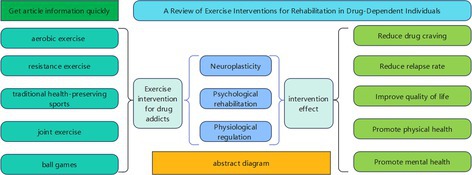

## 1 Introduction

Guided by the core principles of ‘prioritizing prevention, scientific rehabilitation, comprehensive correction, humanistic care, and social collaboration’ as stipulated in the Anti‐Drug Law of the People's Republic of China and the Regulations on Drug Rehabilitation, China's anti‐drug and rehabilitation initiatives have developed in a sustained and institutionalized manner. Globally, the nonmedical use of narcotic drugs has escalated into a major public health crisis requiring urgent intervention, posing serious threats to multiple dimensions of public health, while drug addiction and substance abuse continue to undermine social stability and public order. By the end of 2023, driven by the worsening global drug landscape, China witnessed a renewed surge in drug‐related offences. The number of registered drug users reached 896,000, representing approximately 6.4% of the total national population, signalling a re‐emergence of drug‐related challenges requiring coordinated national and international responses [1].

Drug addiction is a prevalent, relapsing and chronic brain disorder resulting from prolonged substance abuse, primarily marked by neurobiological alterations within the central nervous system. A defining feature of this condition is the progressive loss of voluntary control over drug intake, whereby individuals become increasingly incapable of resisting intense drug cravings. This dysregulation culminates in compulsive and uncontrolled drug‐seeking and drug‐taking behaviours, despite adverse consequences. [2] Drug addiction is a prevalent, relapsing chronic brain disorder resulting from substance abuse, characterized by damage to the central nervous system due to repeated drug use. The hallmark of this disorder is manifested in three progressive stages, each marked by escalating alterations in brain function and behaviour [3]. Conversely, narcotics inflict irreversible and extensive damage on various tissues and organs in the human body, while simultaneously inducing persistent psychological dependence. Exercise has been scientifically demonstrated to significantly reduce craving intensity in individuals with SUD, establishing it as an evidence‐based adjunctive therapy for relapse prevention within comprehensive treatment protocols [4]. Exercise‐based rehabilitation has emerged as a primary development pathway in eco‐friendly rehabilitation, owing to its inherent safety, cost‐effectiveness, broad applicability and sustainable outcomes. The implementation of exercise‐based rehabilitation protocols not only aligns with President Xi Jinping's directives on rehabilitation efforts but also represents an innovative step forward in presenting a ‘Chinese approach’ to global drug governance. This initiative directly responds to the strategic vision set forth by the Ministry of Justice, which emphasizes ‘prioritizing exercise‐based rehabilitation as the foundation for establishing a Chinese‐model rehabilitation system,’ marking a critical step toward advancing rehabilitation practices in a scientifically grounded and specialized direction [5]. This comprehensive review aims to synthesize existing literature on exercise‐based drug rehabilitation, explore its underlying mechanisms, elucidate the differential therapeutic effects of various exercise modalities, optimize evidence‐based intervention protocols, and establish a theoretical framework to guide future research in this domain.

## 2 Materials and Methods

We followed the guidelines for retrieving articles, focusing on Arksey and O'Malley's five key stages of the scoping review process in principle, including (1) defining the research question, (2) identifying relevant studies, (3) selecting studies for inclusion, (4) extracting and charting data and (5) synthesizing and summarizing findings. The detailed report is as follows.

### 2.1 Literature Retrieval and Inclusion and Exclusion

The literature search was primarily conducted by Wang Zhaosong and Wang Hao, who independently carried out literature screening. Disagreements were resolved by their supervisor, He Qinghua. Cohen's κ coefficient was employed to evaluate the consistency of the search results. The final κ value was 0.76 (95% CI: 0.68–0.84, *p* < 0.001), indicating a high level of consistency in the screening results. A total of 1415 core articles were retrieved from both Chinese and English databases. After removing 446 duplicate articles, 802 articles were manually excluded by reading their titles, abstracts and keywords. Seventy‐three articles for which full texts could not be obtained, and 51 articles that were noninterventional, had incomplete results, or were unclear, were excluded. Among the remaining 43 articles, there were disputes regarding the inclusion of five articles between Wang Zhaosong and Wang Hao, and these were ultimately reviewed and excluded by Dr. He Qinghua. One additional article was retrieved using other methods, resulting in a final total of 39 articles included in the study.

**FIGURE 1:**
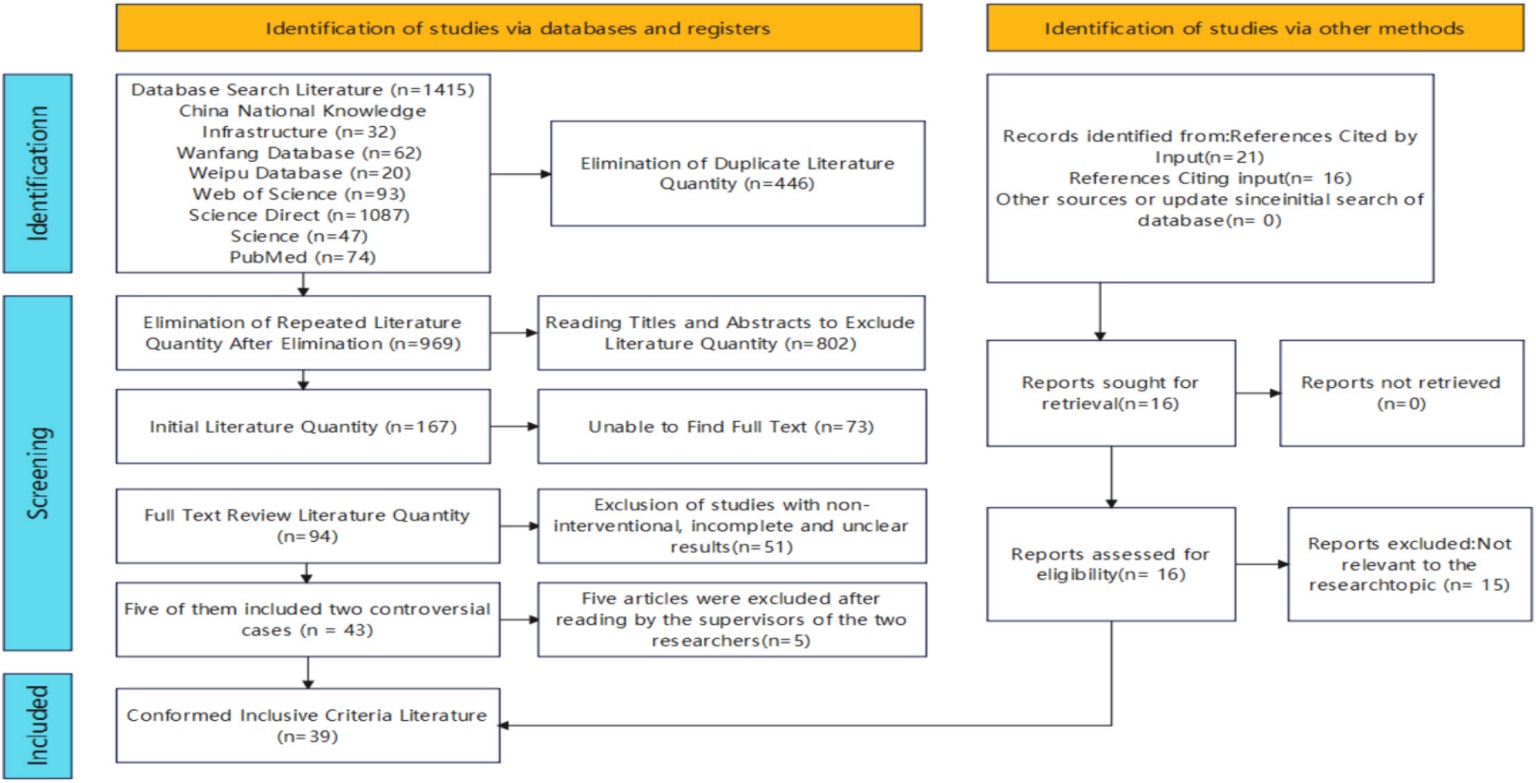
Flow chart of inclusion and exclusion of literature retrieval.

The specific search methods are as follows: Using the Chinese database China National Knowledge Infrastructure (CNKI), the search terms were set as the subject: (Exercise intervention AND drug rehabilitation) OR (Aerobic exercise OR resistance training AND drug rehabilitation) OR (Traditional exercise AND drug rehabilitation AND mental health) OR (Exercise intervention AND drug rehabilitation AND relapse) OR (Exercise intervention AND pharmacological treatment AND drug rehabilitation efficacy). The literature type was set as core journals, and the search time range was from January 1, 2015, to May 20, 2025. A total of 32 relevant articles were retrieved. Through the Chinese database Wanfang Data, the search terms were set as the subject: (Exercise intervention AND drug rehabilitation) OR (Aerobic exercise OR resistance training AND drug rehabilitation) OR (Traditional exercise AND drug rehabilitation AND mental health) OR (Exercise intervention AND ‘drug rehabilitation AND relapse’) OR (Exercise intervention OR pharmacological treatment AND drug rehabilitation efficacy). The literature type was set as core journals, and the search time range was from January 1, 2015, to May 20, 2025. A total of 62 relevant articles were retrieved. In the Chinese database VIP (Chinese Scientific Journal Database), the search terms were set as the subject: (Exercise intervention AND drug rehabilitation) OR (Aerobic exercise AND resistance training AND drug rehabilitation) OR (Traditional exercise AND drug rehabilitation AND mental health) OR (Exercise intervention AND drug rehabilitation OR relapse) OR (Exercise intervention AND pharmacological treatment OR drug rehabilitation efficacy). The literature type was set as core journals, the search time range was from January 1, 2015, to May 20, 2025, the search mode was professional search, and the results were filtered to include only those with full texts. A total of 20 relevant articles were retrieved. Using the English database Web of Science, the search terms were set as the subject: ((Exercise OR exercise therapy OR physical activity OR sport OR ‘movement‐based intervention’) AND (‘drug addiction OR substance abuse OR narcotic abstinence OR withdrawal syndrome)). The literature type was set as Article, the time span was from 2015 to 2025, and the indexes were SCI‐EXPANDED and SSCI. The refinement was based on open access, and a total of 93 relevant articles were retrieved. In the English database ScienceDirect, the search terms were set as the subject: ((exercise OR physical activity OR sport) AND (drug addict OR substance abuse OR narcotic abstinence)). The literature type was set as Journal, the time span was from 2015 to 2025, and the access type was Open Access. A total of 1,087 relevant articles were retrieved. Through the English database PubMed, the search terms were set as the subject: (((Exercise[Mesh]) OR Exercise Therapy[Mesh] OR Sports’[Mesh]) AND (Substance‐Related Disorders[Mesh] OR Drug Users[Mesh] OR Substance Abuse Treatment[Mesh])). The publication time was from January 1, 2015, to May 20, 2025, the article types were Clinical Trial, Meta‐Analysis, and Randomized Controlled Trial, and full texts were required to be freely available. A total of 74 relevant articles were retrieved. Using the database Science, the search terms were set as the subject: (exercise intervention OR physical activity) AND (drug dependence OR addiction recovery). The time range was set as the last 10 years, and the content type was Research Articles. A total of 47 relevant articles were retrieved. As shown in Figure 1.

### 2.2 The Assessment Results of the Methodological Quality and Risk of Bias of the Included Studies Are Presented (See Table 2 and Figure 2)

**FIGURE 2:**
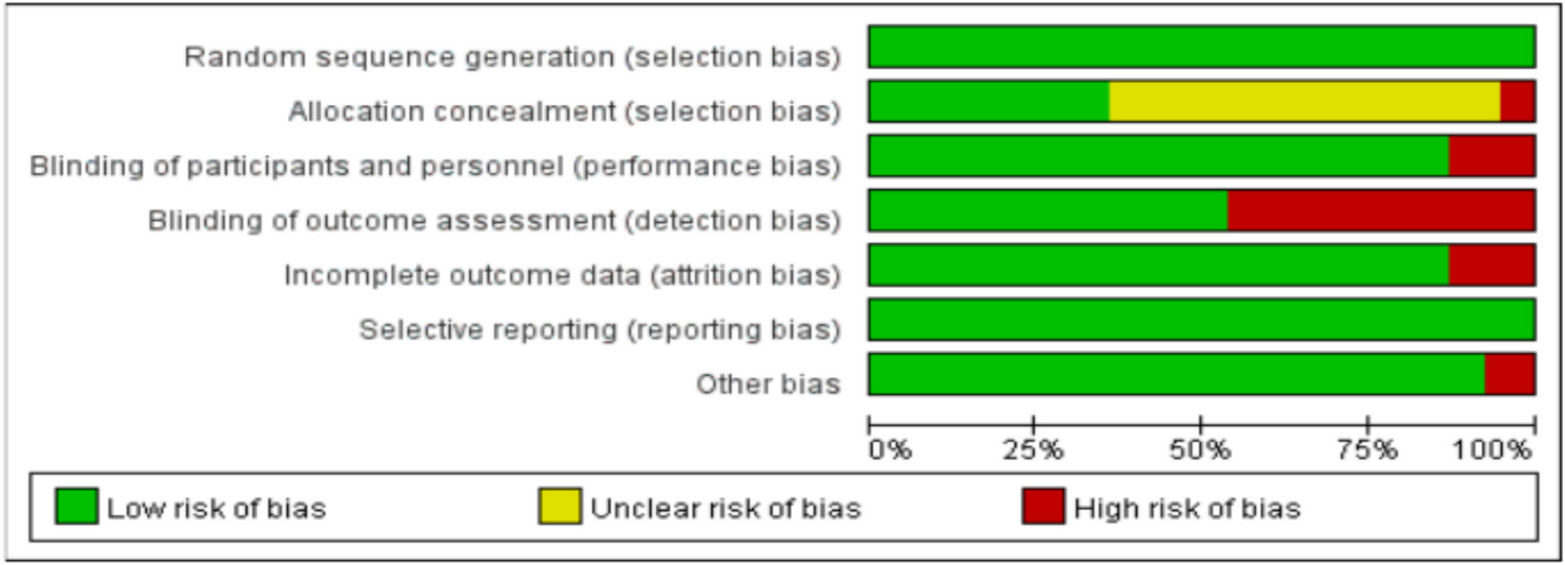
The overall assessment of risk of bias.

We utilized the bias risk assessment tool in RevMan to evaluate the bias risks of the included studies. The assessment results indicated that, in the pooled studies, there was a high risk of bias regarding participant blinding (implementation bias), allocation concealment (selection bias), outcome assessor blinding (detection bias), as well as other sources of bias, while the risks associated with some biases remained unclear. In contrast, the pooled studies demonstrated a low risk of bias in terms of incomplete data (attrition bias), selective reporting (reporting bias) and random sequence generation (selection bias) Table 1.

**TABLE 1:** Risk of bias within studies included in the meta‐analysis.

Included studies	Random sequence generation	Allocation concealment	Blinding of participants and personnel	Blinding of outcome assessment	Incomplete outcome data	Selective reporting	Other bias
Chen Yifan [15]	Yes	Unknown	Yes	Yes	No	None	None
Ding Zenghui [22]	Yes	Unknown	No	Yes	Yes	None	None
Dong Guijun [26]	Yes	Unknown	Yes	Yes	No	None	None
Fu Guangjian [27]	Yes	Unknown	No	No	No	None	None
Geng Jingjing [28]	Yes	Unknown	Yes	No	No	None	None
Guo Yin [29]	Yes	Unknown	Yes	No	No	None	None
Jia Dongming [30]	Yes	Unknown	Yes	No	No	None	None
Liang Xueping [31]	Yes	Unknown	Yes	No	No	None	None
Li Dan [32]	Yes	Unknown	Yes	No	No	None	None
Li Hong [17]	Yes	Yes	No	No	No	None	None
Li Kefeng [33]	Yes	Unknown	Yes	No	Yes	None	None
Li Ning [13]	Yes	Unknown	Yes	No	No	None	None
Li Songyang [34]	Yes	Yes	Yes	No	No	None	None
Liu Yang Zichun [8]	Yes	Yes	Yes	Yes	Yes	None	None
Lu Chunxia [7]	Yes	Unknown	Yes	No	No	None	None
Lu Chunxia [35]	Yes	Unknown	Yes	Yes	No	None	None
Lu Chunxia [36]	Yes	No	Yes	Yes	Yes	None	None
Lu Zhaohui [5]	Yes	Yes	Yes	Yes	No	None	None
Meng Linsheng [24]	Yes	No	Yes	No	No	None	None
Niu Zhong [37]	Yes	Unknown	Yes	Yes	No	None	None
Shen Menglu [23]	Yes	Unknown	Yes	Yes	No	None	None
Song Liangyuan [10]	Yes	Unknown	Yes	Yes	No	None	None
Wang Dongshi [20]	Yes	Yes	Yes	Yes	No	None	None
Wang Jingsong [19]	Yes	Yes	Yes	Yes	No	None	Present
Wang Kun [9]	Yes	Yes	Yes	Yes	No	None	Present
Wang Kun [38]	Yes	Unknown	Yes	Yes	No	None	None
Wang Mu [39]	Yes	Yes	No	Yes	No	None	None
Wei Meiqi [21]	Yes	Yes	Yes	No	No	None	None
Wu Lijun [40]	Yes	Unknown	Yes	Yes	No	None	None
Yang Xinghua [25]	Yes	Yes	No	Yes	No	None	None
Yao Hui [41]	Yes	Yes	Yes	No	No	None	None
Zhai Xiaohui [14]	Yes	Unknown	Yes	No	No	None	None
Zhang Ling [42]	Yes	Unknown	Yes	Yes	No	None	None
Zhao Qi [11]	Yes	Unknown	Yes	No	No	None	None
Zhao Qi [12]	Yes	Unknown	Yes	No	No	None	None
Zhao Zhenhu [18]	Yes	Unknown	Yes	Yes	No	None	None
Zheng Zhijun [6]	Yes	Yes	Yes	Yes	No	None	None
Zhu Dong [43]	Yes	Yes	Yes	No	Yes	None	None
Zhu Yuping [16]	Yes	Yes	Yes	Yes	No	None	Present

## 3 The Biological Mechanism of Drug Addiction

### 3.1 Concept Definition

Drug addiction refers to the compulsive acquisition and use of a specific substance by any means necessary, while disregarding the negative consequences [44]. In their research on advancements in drug addiction, Li Longhui et al. outlined the following eight diagnostic criteria for drug addiction: craving, tolerance, withdrawal, compulsivity, impulsivity, priority, intentionality and persistence [45]. Drug addiction manifests as a compulsive urge to misuse addictive substances for nonmedical purposes, typically characterized by escalating dosage and frequency of use. Tolerance develops as the body physiologically adapts to the drug, while withdrawal symptoms emerge upon cessation or reduction of use. These symptoms may be alleviated or eliminated once the substance is discontinued. Compulsivity is reflected in the impaired control over the initiation, termination and dosage of substance use, often disregarding societal and legal constraints and exhibiting diminished self‐regulation. Priority refers to the increasing neglect of previously important activities, resulting in the disruption of familial and social relationships. Intentionality describes the continued abuse of the substance despite awareness of the harm caused by addiction, while persistence refers to the resurgence of withdrawal symptoms after a period of abstinence and the increase in dosage upon relapse. Under China's Criminal Law, drugs are explicitly defined to include opium, heroin, methamphetamine (crystal meth), morphine, cannabis, cocaine and other addiction‐inducing narcotics or psychotropic substances regulated by national legislation [46].

### 3.2 Neurotransmitters and Drug Addiction

Dopamine, a monoamine neurotransmitter, has been implicated in the underlying mechanisms of drug addiction, with recent research suggesting its role in mediating addiction through the mesocorticolimbic dopamine system [47, 48]. The mesocorticolimbic system is primarily composed of key structures, including the ventral tegmental area (VTA) and the nucleus accumbens (NAc). Within the VTA, dopaminergic neurons make up over 60% of its cellular population and project densely to the NAc, forming a critical neural pathway [49]. Dopamine, a neurochemical that regulates multiple physiological responses within the central nervous system, constitutes the mesolimbic dopamine pathway—the brain's primary reward circuitry. This system not only mediates drug‐induced positive reinforcement and associative learning but also triggers persistent cravings, which ultimately drive addictive behaviours. Simultaneously, endorphins serve as key neurotransmitters in the addiction circuit by: (1) modulating reward processing through binding to specific brain receptors, thereby enhancing pleasurable sensations; (2) influencing cognitive functions, including thought processes, memory and learning; and (3) regulating behavioural motivation. Together, endorphins orchestrate neurotransmitter release dynamics, driving intense behavioural dependencies and pathological cravings [50]. Dopamine, acting as a direct reward neurotransmitter in drug addiction, induces abnormalities in affective states (e.g., euphoria) and behavioural patterns (e.g., impulsivity) through its binding to dopamine receptors [51]. This is one of the primary mechanisms underlying drug addiction.

### 3.3 Brain Structure and Drug Addiction

Research has demonstrated that the primary neuroanatomical substrates of drug action are concentrated in key brain regions, including the VTA, prefrontal cortex (PFC), NAc, olfactory tubercle (OT), amygdala, lateral septal nucleus (LSN) and hippocampus [52]. From a neuroanatomical perspective, the development of drug dependence involves intricate interactions among these regions, which form the neurobiological basis of addiction. These interconnected structures collectively modulate behavioural patterns and physiological responses. Moreover, core neural pathways—specifically the nigrostriatal, mesolimbic and mesocortical pathways—play critical roles in the pathophysiological process of substance dependence. These circuits, beyond regulating normal neurophysiological functions, undergo neuroadaptive changes in response to drug exposure. Aberrant activity within these pathways drives intense drug cravings and compulsive drug‐seeking behaviours, ultimately exacerbating the severity of addiction [53].

### 3.4 Epigenetics and Drug Addiction

Drug use not only induces drug sensitization but also exerts specific stimulatory effects on the nervous system. To a certain extent, addiction can be conceptualized as a disorder of learning and memory functions. As such, the long‐term neural mechanisms underlying addiction‐related memories remain a critical challenge and focal point in addiction research. Recent studies suggest that persistent maladaptive behavioural patterns in drug addiction may result from long‐term transcriptional changes mediated by epigenetic regulation [54]. As research delves deeper into the molecular mechanisms that underpin the persistence of addiction memories, epigenetic modifications have emerged as a key focus, offering a promising new paradigm for the investigation of substance addiction [55]. Mounting evidence indicates that epigenetic regulation of gene expression—particularly through the dynamics of histone acetylation and deacetylation—plays a pivotal role in the progression of addictive behaviours by facilitating neuroadaptations associated with drug reward pathways, which are implicated in SUD [56, 57]. Addicts exhibit significant alterations in epigenetic factors, which substantially accelerate the development of drug addiction, a persistent pathology that is notoriously difficult to reverse. Using advanced epigenetic techniques, researchers have enhanced the binding between the epigenetic enzyme Histone Deacetylase 5 (HDAC5) and DNA in experimental animal brains. This breakthrough successfully identified HDAC5 target genes that are critically involved in early‐stage addiction mechanisms. These genes represent promising therapeutic targets for clinicians in the development of innovative treatments or pharmaceuticals aimed at reducing relapse risk in drug‐dependent individuals. These findings suggest that epigenetically oriented interventions could potentially alleviate, and even reverse, the addictive process [58]. Exercise aids individuals with substance addiction in achieving abstinence by exerting multimodal neurobiological effects—modulating neurotransmitter systems, enhancing synaptic plasticity, optimizing intracellular signalling cascades, upregulating neurotrophins, promoting neurogenesis, and mediating epigenetic regulation. These mechanisms collectively strengthen neural resilience against addictive substances, reduce the intensity of drug cravings, and prevent relapse [59].

### 3.5 Neuroplasticity and Drug Addiction

Research dating back to 2010 has demonstrated that addictive substances induce profound synaptic reorganization within limbic brain regions, particularly in the NAc, thereby altering reward circuitry at the structural level [60]. Russo and colleagues have elucidated the correlation between substance addiction and specific neural adaptations, establishing the need for future research to focus on how addictive drugs induce structural and physiological alterations in neurons. Recent studies further reveal that neurons within the reward circuitry undergo significant drug‐induced transformations, not only accelerating neuroplastic reorganization and degeneration but also intensifying neuronal dependence on addictive substances [61]. Building upon these established neuroadaptations, sustained exposure to addictive substances induces maladaptive neuronal remodelling, driving pathological dependence that manifests as SUD. Conversely, exercise enhances glutamatergic neurotransmission within the PFC, improving synaptic efficacy to restore executive control functions—thereby effectively mitigating compulsive drug‐seeking behaviours through structural and functional neuroplasticity [62]‌.Graded resistance exercise regimens have been shown to effectively modulate neurotransmitter systems in individuals with SUD, recalibrating neuroplasticity and neuroadaptation pathways to reduce cue‐elicited craving intensity [40].

### 3.6 Drug Withdrawal and Relapse Mechanism

Research in substance addiction reveals a consistent phenomenon: during early abstinence, individuals exhibit a significantly diminished capacity to experience drug‐induced euphoria. This anhedonic state gradually improves over time; however, once hedonic responsiveness is restored to a critical threshold, intense drug craving may re‐emerge—triggering compulsive drug‐seeking behaviours and resulting in relapse episodes [63]. Relapse episodes involve the coordinated dysregulation of multiple neurotransmitter and hormonal systems. Specifically, cue‐induced relapse, driven by the convergence of amygdaloid complex‐mediated affective dysregulation and hippocampal maladaptive associative learning, represents a key reinstatement pathway in SUD [64, 65]. A major challenge in addiction therapeutics lies in developing phase‐specific interventions during withdrawal that can effectively suppress the intensity of drug cravings, thus achieving complete suppression of relapse behaviours. This represents a critical frontier in addiction research, requiring urgent translational solutions.

## 4 Psychological Mechanism of Drug Addiction

Substance addiction manifests not only as profound physiological and psychological dependence but also through a constellation of somatic and neuropsychiatric comorbidities, including emergent anxiety disorders, major depressive episodes, insomnia‐spectrum sleep disturbances, psychosis‐like manifestations, debilitating withdrawal syndromes and persistent drug cravings, all of which collectively define the addiction phenotype [66]. Neuropsychopathological disruption is characterized by persistent alterations in brain structure and function, wherein chronic drug exposure induces neuronal injury, neurotransmitter dysregulation and maladaptive synaptic plasticity. These neuroadaptations simultaneously impair emotional regulation circuits, compromise executive control networks and potentiate aggression‐primed maladaptive coping strategies during conflict processing, thereby establishing a self‐perpetuating cycle of addiction‐related neuropathology [67]. According to the framework of negative reinforcement in emotional processing, depressive and anxious states are considered primary triggers for individuals with addiction to experience intense cravings for drugs. During the detoxification process, addicts often undergo a series of unpleasant emotional experiences, which become a heavy burden they seek to escape. In an attempt to alleviate these negative emotions, addicts may develop a strong urge to relapse, seeking to reduce their discomfort through drug consumption [68].

## 5 Theoretical Basis of Drug Rehabilitation Through Sports

Within the negative reinforcement framework of affect dysregulation, depressive and anxious states serve as core affective drivers of pathological drug craving. During abstinence, addicts experience an aversive emotional burden characterized by persistent negative affect, which generates a compelling motivation to alleviate distress through relapse, thereby establishing drug readministration as a maladaptive emotional escape mechanism [63]. Empirical studies reveal that while structured exercise regimens have yet to be formally integrated into standardized relapse prevention protocols, converging clinical evidence demonstrates their clinically significant benefits in mitigating relapse trajectories among abstinent individuals [59, 69]. Exercise training provides multidimensional benefits for individuals with SUD: studies show that structured physical activity enhances subjective well‐being, self‐confidence and self‐esteem, while alleviating negative affect. It optimizes prefrontal cognitive control functions, strengthening resistance to drug cues and effectively supporting rehabilitation efforts. Crucially, exercise induces multisystem physiological adaptations, involving respiratory, cardiovascular, neuroendocrine, gastrointestinal and metabolic regulatory pathways [70, 71]. These adaptations contribute to the restoration of organ function, leading to improvements in both physiological and psychological well‐being. Chronic drug abuse typically results in persistent physiological sequelae, including dizziness, cephalalgia and somatic pain, which are often accompanied by affective instability, anxiety–depression comorbidity and impairments in social functioning, including dysfunctional communication. In severe cases, violent behaviours or suicidal ideation may emerge. These multidimensional pathologies not only devastate individual health but also pose significant societal risks [72]. Exercise interventions activate immuno‐inflammatory responses and modulate dopaminergic neurotransmission, significantly alleviating depression and anxiety symptoms in abstinent individuals. This reduction in negative affect enhances overall mental health, and crucially, these interventions reduce psychological dependence on substances and diminish drug craving symptoms—representing essential neurobiological mechanisms for sustained recovery and relapse prevention [42].

## 6 Research Progress of Drug Rehabilitation by Exercise Intervention

### 6.1 Analysis of Examples of Aerobic Exercise for Drug Rehabilitation

Analysis of Table 2 reveals a range of aerobic exercise interventions for drug rehabilitation, including running, cycling, rope skipping, callisthenics, workplace exercises, aerobic dance, rehabilitation‐specific callisthenics and high‐intensity interval training (HIIT). These interventions demonstrate multidimensional benefits: Zheng Zhijun et al. established that moderate‐to‐vigorous intensity exercise significantly alleviates anxiety and depression, while reducing drug craving among abstinent individuals. Physiologically, Wang Kun et al. demonstrated that moderate‐intensity aerobic dance improves body composition parameters—such as reduced BMI and body fat percentage—in female methamphetamine users. In terms of relapse prevention, Zhao Qi et al. reported that combining callisthenics with cycling training effectively suppresses cue reactivity in methamphetamine addicts. For cognitive enhancement, Li Ning et al. showed that HIIT optimizes autonomic regulation, as evidenced by increased heart rate variability (HRV). Wang Kun et al. further validated that mindfulness‐integrated aerobic dance prolongs slow‐wave sleep duration. Cardiovascular improvements were confirmed by Wei Meiqi et al., who noted superior blood pressure and lipid modulation with higher‐intensity regimens. Additionally, Chen Yifan et al. found that regular aerobic exercise not only reduces cravings but also enhances social adaptability through working memory enhancement. Collectively, these findings provide a scientific rationale for the integration of nonpharmacological rehabilitation protocols.

**TABLE 2:** Progress in research on aerobic exercise for drug rehabilitation (*n* = 20).

Author (year)	Sample source	Sample size	Participants	Intervention approach	Exercise frequency (time)	Movement intensity	Intervention duration	Physiological and psychological improvement effect
Zheng Zhijun, 2016 [6]		IG: 60 CG: 60	‌MA	Running, power cycling, jumping rope, aerobics	3–4 times per week 30–90 min	Moderate to high intensity (64%–80% HRmax)	Four months	Most factors of SCL‐90 (*p* < 0.01), depression (*p* < 0.05) and paranoia (*p* < 0.05)
Lu Chunxia, 2023 [7]	DRC	IG: 61 CG: 31	‌MA	Workout, treadmill	5 times per week 80 min	Moderate to high intensity(60%–85% HRmax)	Three months	Improvement of depression, mental health, dependence and prevention of relapse (*p* < 0.01)
Liu Yangzichun 2021 [8]	DRC	IG: 54 CG: 54	SD	Flexibility, strength, endurance,	3 times per week 60 min	High intensity intervals(70%–80% HRmax)	Four months	Body mass, BMI and body fat decreased (*p* < 0.01), serotonin and dopamine increased (*p* < 0.01, *p* < 0.05) and brain‐derived neurotrophic factor and cortisol decreased (*p* < 0.05)
Wang Kun, 2022 [9]	DRC	IG: 18 CG: 37	MA	Fit aerobics	3 times per week 60 min	Medium to high intensity(65%–85% HRmax)	Three months	Significantly reduced depression, anxiety and drug craving (*p* < 0.001) and increased VO2max (*p* < 0.001)
Song Liangyuan 2023 [10]	DRC	IG: 36 CG: 12	SD ‌MA	Aerobic exercise combined with mindfulness	3 times per week 40 min	Medium intensity(60%–70% HRmax)	Two months	It had a positive effect on the relapse tendency and negative emotions of addicts (*p* < 0.01)
Zhao Qi, 2022 [11]	DRC	IG: 44 CG: 20	‌MA	Aerobic exercise combined with mindfulness	3 times per week 40 min	Medium intensity(65%–70% HRmax)	Two months	Reduced MA attention bias (*p* < 0.001)
Zhao Qi 2024 [12]	DRC	IG: 42 CG: 21	‌MA	Dance, cycling	3 times per week 40 min	Medium intensity(65%–70% HRmax)	Three months	Enhance attentional inhibition in female MA addicts (*p* < 0.01).
Li Ning 2024 [13]	DRC	IG: 30 CG: 24	‌MA	Intermittent aerobic exercise	3 times per week 40–45 min	High intensity(75%–85% HRmax)	Three months	Reduce blood pressure, heart rate variability and respiratory function in MA addicts (*p* < 0.01).
Zhai Xiaohui 2025 [14]	DRC	IG: 36 CG: 32	‌MA	Acute aerobic exercise	3 times per week 30 min	Medium intensity(70%–80% HRmax)	Three months	Enhance cognitive function and reduce relapse in addicts (*p* < 0.05).
Chen Yifan 2021 [15]	DRC	IG: 38 CG: 19	‌MA	aerobic exercise	2–3 times per week 30 min	Medium and high intensity(65%–85% HRmax)	Three months	Improve drug craving and working memory in addicts (*p* < 0.01).
ZhuYuping 2023 [16]	DRC	IG: 32 CG: 32	‌MA	Bicycles, running	3 times per week 60 min	Medium intensity(65%–75% HRmax)	Two months	Improve working memory and inhibitory control in MA‐dependent individuals (*p* < 0.05).
Li Hong, 2024 [17]	DRC	IG: 148	SD	Brisk walking	3 times per week 60 min	Medium intensity(65%–70% HRmax)	Three months	Improvement of depression, sleep quality and drug craving in drug addicts (*p* < 0.05)
Zhao Zhenhu 2017 [18]	DRC	IG: 30 CG: 30	SD	Running, cycling	3 times per week 60 min	Medium intensity(60%–79% HRmax)	Three months	Significant improvements were seen in BMI, lung capacity, sit‐and‐reach test, step index, single‐leg standing and push‐ups (*p* < 0.01), along with notable reductions in SCL‐90 scores for forced state, interpersonal issues, depression, anxiety, hostility, phobia and psychosis (*p* < 0.05).
Wang Jingsong, 2022 [19]	DRC	IG: 22 CG: 21	‌MA	Run	5 times per week 50 min	Medium intensity(64%–76% HRmax)	Three months	MA withdrawal significantly increased VO2max (*p* < 0.05), and decreased cholesterol, triglyceride, blood glucose, waist circumference, body fat percentage, triglyceride and blood glucose (*p* < 0.05)
Wang Dongshi, 2017 [20]	DRC	IG: 26 CG: 27	MA	Powerbike, jogging, jumping rope	3 times per week 30–40 min	Medium intensity(65%–75% HRmax)	Three months	Reduced anxiety and depression in MA‐dependent patients (*p* < 0.05) and increased VO2max (*p* < 0.01)
Wei Meiqi, 2024 [21]	DRC	IG: 64 CG: 31	SD ‌MA	Volleyball, cycling	7 times per week 45 min	Different intensity(40%–85% HRmax)	Six months	Relapse prevention, lung capacity, sleep quality and reaction time were significantly improved (*p* < 0.05)
Ding Zenghui, 2021 [22]	DRC	IG: 55 CG: 47	SD	Gymnastics	10 times a week 40 min	Medium intensity(65%–75% HRmax)	Eight months	Fat loss, prevention of osteoporosis (*p* < 0.05)
Shen Menglu, 2021 [23]	DRC	IG: 160 CG: 80	‌MA	HIIT training, MICT training	3 times per week 24–60 min	Medium intensity(70%–80% HRmax), high strength (80%–90% HRmax)	Six months	Reduce drug craving and improve memory and executive function (*p* < 0.05).
Meng Linsheng, 2023 [24]	DRC	IG: 30 CG: 30	SD	HIIT training	3 times per week 45 min	High intensity(75%–85% HRmax)	Four months	Lower blood pressure, reduce fat and increase muscle, improve respiratory function(P<0.05)
Yang Xinghua, 2020 [25]	DRC	IG: 47 CG: 47	MA	‌Aerobics	3 times per week 40 min	Medium intensity(65%–75% HRmax)	12 months	Increase the success rate of detoxification and improve the quality of life for individuals undergoing drug rehabilitation (*p* < 0.05).

*Note:* The significance threshold was set at *p* = 0.05, and the confidence intervals were calculated at the 95% level.

Abbreviations: CG = control group; DRC = drug rehabilitation centre; IG = intervention group; MA = methamphetamine; SD = synthetic drugs.

### 6.2 Analysis of Antiresistance Exercise Detoxification Cases

Both studies in Table 3 implemented resistance training interventions, demonstrating significant efficacy in enhancing both physiological and psychological health parameters among individuals with SUD. Wu Lijun et al. employed daily variable‐intensity resistance training, yielding optimal outcomes, while Li Kefeng et al. utilized longer‐duration interventions with thrice‐weekly moderate‐intensity protocols. Crucially, both studies converged in demonstrating resistance training's capacity to: 1) optimize neurotransmitter systems, 2) promote neuroadaptive changes, 3) reduce the intensity of drug cravings and 4) achieve improvements in emotional regulation, sleep architecture and overall psychological status. These findings collectively suggest that resistance training induces more pronounced neurological outcomes in addiction rehabilitation.

**TABLE 3:** Research progress of resistance exercise for drug rehabilitation (*n* = 2).

Author (year)	Sample source	Sample size	Participants	Intervention approach	Exercise frequency (time)	Movement intensity	Intervention duration	Physiological and psychological improvement effect
Wu Lijun 2023 [40]	DRC	IG: 60 CG: 30	Heroin	Resistive exercise	7 times per week 30 min	Different intensity(60%–85% HRmax)	Two months	Increased concentrations of dopamine, β‐endorphin, serotonin and brain‐derived neurotrophic factor Fos family proteins in addicts, and decreased visual analogue scores for craving (*p* < 0.05)
Li Kefeng 2021 [33]	DRC	IG: 26	‌MA	Resistive exercise	3 times per week 24 min	Medium intensity(65%–75% HRmax)	Four months	Improved sleep, anxiety, depression and thirst (*p* < 0.001). Oxygenated haemoglobin concentrations in the left prefrontal cortex, right prefrontal cortex and left motor cortex were significantly increased (*p* < 0.001)

*Note:* The significance threshold was set at *p* = 0.05, and the confidence intervals were calculated at the 95% level.

Abbreviations: CG = control group; DRC = drug rehabilitation centre; IG = intervention group; MA = methamphetamine; SD = synthetic drugs.

### 6.3 Analysis of Traditional Health Care Sports and Drug Rehabilitation Cases

Analysis of Table 4 indicates a variety of traditional wellness exercises—including Tai Chi, Tai Chi rehabilitation exercises, yoga, aerobic callisthenics, meditation, physical training and One‐Finger Zen Tuina—each employing moderate intensity, highlighting the versatility of exercise interventions in addiction recovery. Geng Jingjing's research confirmed that 3 months of moderate‐intensity Tai Chi significantly enhances psychological health and reduces systolic blood pressure in abstinent individuals. Zhu Dong established that the 24‐form Tai Chi improves rehabilitation success rates. Yao Hui demonstrated that yoga and meditation significantly lower kynurenine levels in female addicts, mitigate relapse risk, and enhance physical capacity. Wang Mu's experiment revealed that Tai Chi effectively reduces drug craving intensity among female participants. Zhang Ling showed that running and Tai Chi ameliorate attentional bias and shorten reaction times. Jia Dongming documented the efficacy of Baduanjin in reducing body fat and improving the quality of life in overweight addicts. Fu Guangjian highlighted the significant improvements brought about by Five‐Animal Exercises in emotional dysregulation and sleep architecture. Collectively, these findings underscore the multidimensional benefits of traditional mind–body practices for both physiological and psychological rehabilitation.

**TABLE 4:** Research progress of traditional health‐preserving sports and drug rehabilitation (*n* = 7).

Author (year)	Sample source	Sample size	Participants	Intervention approach	Exercise frequency (time)	Movement intensity	Intervention duration	Physiological and psychological improvement effect
Geng Jingjing 2016 [28]	DRC	IG: 30 CG: 27	SD	‌Tai Chi‌	5 times per week 45 min	Medium intensity(65%–75% HRmax)	Three months	SCL‐90, blood pressure reduction (*p* < 0.01), balance ability (*p* < 0.05)
Zhu Dong 2018 [43]	DRC	IG: 42 CG: 33	SD	Form 24 Tai Chi	3–5 times per week 55 min	Medium intensity(65%–75% HRmax)	Six months	Number of days of abstinence, number of days of abstinence after discharge, number of days of abstinence without discovery (*p* < 0.01)
Liang Xueping 2019 [31]	DRC	IG: 36 CG: 37	SD	Yoga, meditation	3 times per week 40 min	Medium intensity(65%–75% HRmax)	Three months	Quality of life, depression, anxiety (*p* < 0.05)
Wang Mu 2022 [39]	DRC	IG: 48 CG: 47	SD	‌Tai Chi	10 times per week 30 min	Medium intensity(65%–75% HRmax)	Three months	Reduce drug craving in addicts (*p* < 0.001).
Zhang Ling 2024 [42]	DRC	IG: 44	‌MA	Running, Tai Chi	3 times per week 60 min	medium intensity(65%–75% HRmax)	Three months	There is a significant improvement in both attentional bias and reaction time among addicts (*p* < 0.05).
Jia Dongming 2022 [30]	DRC	IG: 36 CG: 18	SD	Baduanjin	5 times per week 60 min	medium intensity(65%–75% HRmax)	Two months	Improvements were observed in waist circumference, BMI, body weight, hip circumference, body fat percentage, cholesterol levels, triglyceride levels and Visual Analog Scale scores (*p* < 0.05).
Fu Guangjian 2016 [27]	DRC	IG: 100 CG: 100	‌MA	Wuqinxi	7 times per week 30 min	Medium intensity (65%–75% HRmax)	Five months	Anxiety, depression and sleep quality scores were significantly reduced (*p* < 0.05)

*Note:* The significance threshold was set at *p* = 0.05, and the confidence intervals were calculated at the 95% level.

Abbreviations: CG = control group; DRC = drug rehabilitation centre; IG = intervention group; MA = methamphetamine; SD = synthetic drugs.

### 6.4 Analysis of Joint Movement Drug Rehabilitation Cases

Studies in Table 5 universally employed combined aerobic and resistance training, demonstrating the efficacy of integrated exercise modalities in drug rehabilitation. These interventions featured flexible designs, with frequencies of 3–5 sessions per week and durations ranging from 25 to 80 min. Lu Chunxia's research revealed that balance‐aerobic‐resistance training significantly reduced inflammatory markers in amphetamine‐dependent individuals, while also enhancing mental health and reducing drug cravings. Li Songyang confirmed synergistic improvements in both physiological and psychological recovery for methamphetamine addicts. Lu further emphasized that this combination of exercises helps diminish the severity of dependence and strengthens impulse control, thereby reducing the risk of relapse. Guo Yin's work demonstrated that treadmill/elliptical training combined with strength‐endurance exercises optimized plasma oxytocin/vasopressin levels in male addicts, alleviating perceived stress and enhancing fitness metrics. Collectively, these findings validate the multidimensional benefits of hybrid exercise interventions in addiction rehabilitation.

**TABLE 5:** Progress of joint exercise drug rehabilitation research (*n* = 6).

Author (year)	Sample source	Sample size	Participants	Intervention approach	Exercise frequency (time)	Movement intensity	Intervention duration	Physiological and psychological improvement effect
Lu Chunxia 2021 [35]	DRC	IG: 34 CG: 63	MA	Balance, aerobic combined resistance training	5 times per week 25 min	Low to high intensity (60%–80% HRmax)	Three months	Improve inflammatory factors, improve mental health, reduce the degree of drug craving (*p* < 0.05)
Li Songyang 2022 [34]	DRC	IG: 88 CG: 88	‌MA	Aerobic exercise combined Resistance exercise	3 times per week 55 min	Medium intensity (60%–75% HRmax)	Two months	Reduced MA trait anxiety and psychological craving (*p* < 0.01) improved mental health status (*p* < 0.001)
Lu Chunxia 2023 [36]	DRC	IG: 65 CG: 32	‌MA	Aerobic combined resistance	5 times per week 80 min	Medium intensity(57–65 HRmax)	Three months	The expression levels of dopamine and 5‐hydroxytryptamine were significantly increased, while negative emotions and anxiety decreased (*p* < 0.05)
Guo Yin 2021 [29]	DRC	IG: 22 CG: 23	SD	Running and elliptical, strength and endurance training	5 times per week 30 min	Medium intensity(65%–75% HRmax)	Three months	It significantly reduced arginine vasopressin, Self‐Rating Anxiety Scale scores, and Chinese Perceived Stress Scale scores (*p* < 0.01), while increasing oxytocin levels and VO2max (*p* < 0.05).
Wang Kun 2024 [38]	DRC	IG: 32 CG: 16	‌MA	Mindfulness combined with aerobic aerobics group	3 times per week 35 min	Medium intensity(65%–75% HRmax)	Three months	Both sleep quality and drug craving have shown significant improvement (*p* < 0.01).
Yao Hui 2020 [41]	DRC	IG: 30 CG: 10	SD	Yoga, meditation, physical exercise	3 times per week 60 min	Medium and high intensity(60%–85% HRmax)	Three months	Improve the levels of kynurenine and kynurenic acid (*p* < 0.01), and reduce the risk of relapse.

*Note:* The significance threshold was set at *p* = 0.05, and the confidence intervals were calculated at the 95% level.

Abbreviations: CG = control group; DRC = drug rehabilitation centre; IG = intervention group; MA = methamphetamine; SD = synthetic drugs.

### 6.5 Analysis of Drug Rehabilitation Cases in Ball Games

Table 6 analyzes ball sports interventions—specifically basketball, table tennis and badminton—implemented 3 times per week or daily for durations ranging from 20 to 150 min at varied intensities, targeting methamphetamine, heroin and meth users. Lü Zhaohui demonstrated that high‐intensity basketball training accelerates both physiological and psychological rehabilitation. Dong Guijun revealed that medium‐intensity table tennis activates motor and prefrontal cortices, thereby enhancing cognitive control. Li Dan confirmed that badminton improves physical fitness and reduces dependency severity, correlating with increased serum levels of brain‐derived neurotrophic factor (BDNF). Niu Zhong identified badminton's capacity to remodel serum proteomic regulators, particularly increasing MyoD1 synthesis, which ameliorates sarcopenia in heroin users. Collectively, these studies indicate that diverse ball sports significantly enhance physical and mental recovery, cognitive function and fitness metrics. Badminton and table tennis, in particular, have been validated as feasible adjunctive therapies in addiction rehabilitation.

**TABLE 6:** Research progress on drug rehabilitation of ball games (*n* = 4).

Author (year)	Sample source	Sample size	Participants	Intervention approach	Exercise frequency (time)	Movement intensity	Intervention duration	Physiological and psychological improvement effect
Lu Zhaohui 2020 [5]	DRC	IG: 120 CG: 120	MA	Basketball	3 times per week 60 min	Hard intensity(60%–90% HRmax)	Four months	Depression, anxiety and stress (*p* < 0.01)
Dong Guijun 2021 [26]	DRC	14	‌MA	Table tennis	3 times per week 20 min	Medium intensity(65%–75% HRmax)	Two months	The Stroop effect was significantly improved (*p* < 0.01), and the two brain regions of rDLPFC (CH17) and rPMC (CH29) were significantly activated (*p* < 0.05)
Li Dan 2022 [32]	DRC	IG: 154 CG: 62	Heroin、MA	Badminton	3 times per week 150 min	Freedom	Two months	Reduced Brain‐derived neurotrophic factor and blood pressure in addicts (*p* < 0.01), increased VO2max, lung capacity and vertical jump (*p* < 0.01)
Niu Zhong 2025 [37]	DRC	IG: 240 CG: 165	Heroin	Badminton	7 times per week 150 min	Freedom	Three months	Increased relative appendicular skeletal muscle mass, hand grip strength and gait speed; decreased testosterone, melatonin, (*p* < 0.01); reduced Myostatin, interleukin‐17, interleukin‐6, tumour necrosis factor α, TNF‐like weak inducer of apoptosis, C‐reactive protein and malondialdehyde levels (*p* < 0.01).

*Note:* The significance threshold was set at *p* = 0.05, and the confidence intervals were calculated at the 95% level.

Abbreviations: CG = control group; DRC = drug rehabilitation centre; IG = intervention group; MA = methamphetamine; SD = synthetic drugs.

## 7 Discussion

Mechanistic analysis reveals that drug addiction involves dysregulation of neurotransmitters, structural brain alterations, epigenetic modifications and impaired neuroplasticity. Dopamine emerges as a principal research focus, primarily synthesized in the VTA and NAc within the mesolimbic pathway. Concurrently, endogenous opioids exert critical modulatory effects on neural signalling and reward processing. As a result, dopamine plays a central mechanistic role in the neuropathology of substance addiction [47, 48, 49, 50]. Substance addiction is fundamentally rooted in neurobiological substrates involving key brain regions, including the VTA, PFC, NAc, OT, amygdala, LSN and hippocampus. These structurally interconnected regions form integrated neural circuits that mediate drug‐induced neuroadaptive changes. Dysregulation within these pathways drives compulsive drug‐seeking behaviours through maladaptive neuroplasticity [51, 52]. Substance addiction demonstrates significant epigenetic involvement, with research confirming that drug exposure dynamically alters epigenetic regulators. These modifications entrench addiction pathology, intensify withdrawal severity, and impede cessation efforts. Critically, targeting these epigenetic mechanisms represents a pivotal therapeutic strategy for reversing addiction trajectories [53, 54]. Research by Russo, Zhang Wei, Wu Lijun and colleagues demonstrates that substance abuse induces profound neurobiological changes, altering neural structure and physiological functions, which exacerbate addiction and hinder recovery. Engaging in physical activity can improve neurological states and reduce drug cravings. Furthermore, drug addiction is closely linked to psychological factors, creating not only psychological dependence but also physiological symptoms, including anxiety, depression, sleep disturbances and psychiatric abnormalities. Chronic or repeated drug use damages brain neurons, disrupts neurotransmitter balance, and impairs synaptic plasticity, compromising emotional regulation, cognitive function and decision‐making. To escape these adverse states, addicts develop intense cravings to use drugs again, seeking to alleviate negative emotional distress [59, 60].

**FIGURE 3:**
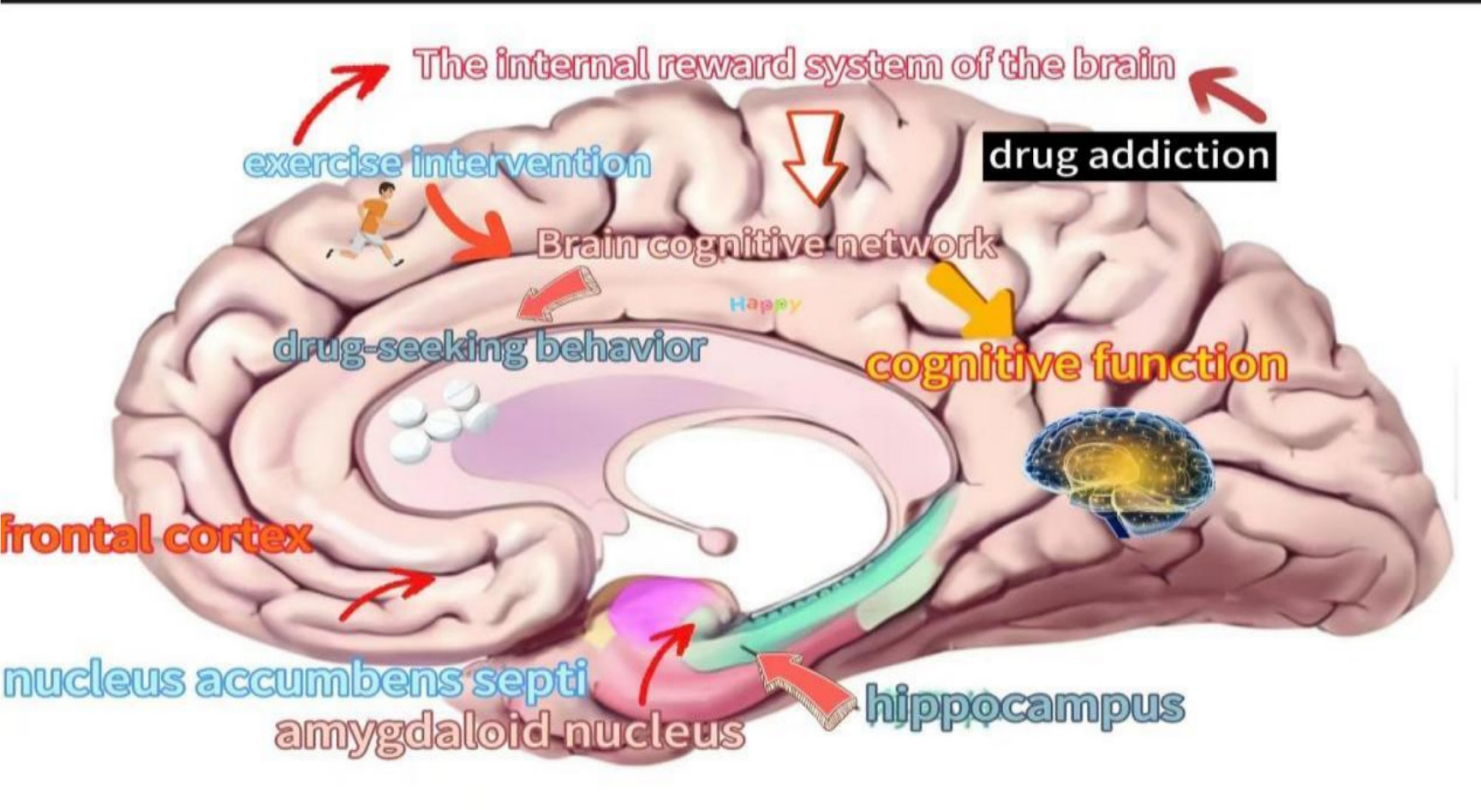
Theoretical model of drug addiction intervention in movement [42].

Figure 3 presents the theoretical model of exercise intervention for drug addiction. Physical activity activates the brain's intrinsic reward neurotransmitter system. Drug addiction typically involves excessive dopamine (DA) secretion within the mesolimbic pathway, which elevates DA levels, inducing euphoric excitation. Exercise counteracts this by engaging analogous neural reward circuits, thereby modulating neurotransmitter transmission to generate exercise‐induced reward effects. Furthermore, exercise promotes neural circuit remodelling through enhanced synaptic connectivity and optimized neurotransmitter release/reuptake dynamics. These neuroadaptations improve cognitive function, executive control and emotional regulation while suppressing drug‐seeking neuronal signalling. Crucially, exercise restores dopaminergic system functionality and normalizes midbrain reward pathways, effectively reducing drug cravings and withdrawal symptoms, thereby improving the quality of life for addicts. Concurrently, exercise stimulates endogenous neurotransmitters, such as endorphins, which exert analgesic and antidepressant effects. This neurochemical adjustment stabilizes emotional states and strengthens self‐regulatory capacity, empowering individuals to resist drug‐related impulses and temptations. Physical exercise interventions for reducing drug addiction and facilitating postwithdrawal recovery primarily include aerobic exercise, resistance training, combined aerobic–resistance training, traditional mind–body exercises (e.g., Tai Chi), specialized detoxification programs and ball sports. Most interventions employ moderate‐intensity protocols with frequencies of ≥ 3 sessions per week, though high‐intensity modalities are occasionally explored. Empirical evidence confirms that all these approaches yield beneficial effects on physiological parameters (e.g., cardiovascular and pulmonary function), psychological well‐being and neurological rehabilitation among addicts.

Given the extensive multisystem damage caused by substance abuse, such as cardiovascular and cardiopulmonary impairments, researchers have categorized exercise‐based interventions into two primary models: single‐modality interventions (including aerobic exercise, resistance training, mind–body exercises and ball sports) and combined‐modality interventions (integrating aerobic and resistance exercises). While all these modalities have been shown to reduce drug dependency and craving, their efficacy varies significantly across different intervention types. Current research focuses on five comparative dimensions: (1) the efficacy of normalizing neurotransmitter levels, (2) the capacity for cardiovascular repair, (3) the alleviation of psychological symptoms, (4) the timelines of neuroplastic adaptation and (5) the outcomes of long‐term relapse prevention.

### 7.1 Aerobic Exercise Enhancing Drug Addicts' Physical and Mental Health

Aerobic exercise positively impacts the physical functions and mental health of drug addicts. Research indicates that aerobic exercise can improve blood pressure, blood sugar, blood lipids, gut microbiota, respiratory function, cardiopulmonary function, osteoporosis and overall physical health. Wang Jingsong found that exercise intensity prescriptions based on the ventilatory threshold and percentage of maximum heart rate can reduce blood sugar and blood lipids in methamphetamine withdrawal patients [19]. Zhu Yuping's research revealed that gut microbiota analysis indicates the modulation of intestinal microbial communities and associated metabolic pathways plays a mediating role in exercise‐induced improvements in cognitive function among methamphetamine‐dependent individuals [16]. Li Ning's clinical study demonstrated that high‐intensity interval aerobic exercise significantly reduced blood pressure levels and enhanced respiratory efficiency among individuals with methamphetamine use disorder (MAUD) [13]. Ding Zenghui's research demonstrates that aerobic gymnastics intervention can serve as an effective clinical strategy for preventing and treating drug‐induced osteoporosis in individuals undergoing drug rehabilitation [22]. Zhao Zhenhu's research confirms that aerobic exercise consistently demonstrates significant efficacy in adiposity reduction, cardiopulmonary enhancement and endurance improvement [18]‌. In terms of psychology, relevant studies have shown that aerobic exercise has a good promoting effect on individual working memory, inhibition ability, cognitive function, sleep quality, depression and anxiety [16]. Empirical findings reveal that aerobic exercise training enhances working memory capacity and inhibitory control in methamphetamine‐dependent individuals, thereby mitigating adverse impacts of addiction on cognitive function. Complementing this, research by Shen Menglu and Zhai Xiaohui demonstrates significant improvements in core cognitive domains—specifically episodic memory consolidation and executive function flexibility—following structured aerobic interventions [14, 23]. Wang Kun and Wei Meiqi's clinical trial demonstrates that structured aerobic exercise substantially enhances sleep architecture normalization in patients with MAUD [9, 21]. Lu Chunxia's research empirically validates that exercise interventions activate neuroimmune regulation and enhance dopaminergic neurotransmission, consequently reducing depressive and anxiety symptomatology [7]. Zhao Zhenhu's randomized controlled trial demonstrated superior efficacy of open‐skill exercise interventions in ameliorating comorbid psychological symptoms among substance use disorder (SUD) patients, with significant reductions observed across multiple psychometric domains [18]. Liu Yangzichun's mechanistic study establishes that HIIT induces favourable neuromodulation of anxiety‐ and depression‐associated neurotransmission, generating significant biopsychological improvements in SUD populations [8].

### 7.2 Resistance Training Effects on Drug Addicts' Muscular and Mental States

Wu Lijun's controlled trial demonstrated that 8‐week resistance training regimens across varied intensity levels consistently ameliorated neurotransmitter dysregulation in heroin abstainers, enhanced neuroplastic adaptation mechanisms, and consequently attenuated drug craving behaviour [40]. Li Kefeng demonstrated that moderate‐intensity resistance training significantly activates the left and right prefrontal cortices in methamphetamine addicts, enhancing connectivity between diverse brain regions, with particular emphasis on strengthening myogenic and neurogenic activities in the parietal motor cortex. This intervention promotes comprehensive involvement of the prefrontal and parietal cortices in motor control, improves associations among the PFC, motor cortex and occipital cortex in heart rate activity, respiratory activity and myogenic activity during the resting state, reduces lateralized brain usage, and strengthens regional synchronization. Consequently, it ameliorates sleep quality, anxiety, depression and craving levels, thereby bolstering the psychological well‐being of individuals undergoing detoxification [49].

### 7.3 Traditional Exercises Boosting Drug Addicts' Physio‐Psychological and Self‐Control

Empirical findings confirm that traditional wellness exercises, such as Tai Chi and Qigong, enhance rehabilitation outcomes for (SUDs) by optimizing psychophysiological regulation and executive control. These exercises offer a low‐cost, low‐risk and accessible intervention suitable for diverse settings (Zhang Ling) [42]‌. Scholars including Geng Jingjing, Cui Zhuo, Yao Hui And Liang Xueping have empirically validated that traditional mind–body exercises—such as Tai Chi, Yoga, Yizhichan (one‐finger meditation) and mindfulness meditation—significantly improve rehabilitation outcomes for SUD [28, 31, 41, 73]. Given these promising results, the current research landscape necessitates expanded investigation into the neurobiological underpinnings of traditional mind–body exercises for psychophysiological rehabilitation in substance use populations.

### 7.4 Ball Games Aiding Drug Addicts' Social, Competitive and Rehabilitation Processes

Research indicates that ball sports, such as basketball, soccer and table tennis, significantly enhance social skills, competitive awareness, self‐control and rehabilitation outcomes for individuals recovering from substance addiction. As team‐based activities, ball sports require participants to collaborate, communicate and interact with teammates, which help rebuild social competence and repair interpersonal relationships damaged by drug abuse, facilitating reintegration into society. The competitive nature of these sports fosters a positive mindset, redirecting focus from drug cravings to athletic achievement and personal growth, thereby promoting healthier life goals and values. Additionally, adherence to game rules reinforces discipline and self‐regulation, helping addicts recognize the importance of following societal norms and maintaining behavioural control, ultimately supporting their commitment to a drug‐free life. Studies, such as those by Dong Guijun on table tennis interventions, further demonstrate cognitive improvements, including enhanced activation of the dorsolateral prefrontal cortex (DLPFC) and improved performance on the Stroop test, highlighting the multifaceted benefits of ball sports in addiction recovery. Specifically, Dong Guijun found that table tennis significantly increased DLPFC activation and the correct rate of the Stroop test in drug addicts, indicating that table tennis can improve cognitive conflict control in METH addicts by enhancing DLPFC activation [26].

### 7.5 Combined Exercise Positive Influence on Drug Addicts' Physical and Mental Well‐being

Research indicates that combining moderate‐intensity aerobic exercise with resistance training provides substantial physiological and psychological benefits for individuals recovering from substance addiction. As demonstrated by Lu Chunxia, this integrated approach reduces the expression of inflammatory factors, thereby alleviating neurological toxicity and withdrawal symptoms. It also concurrently improves negative mood, enhances mental health, and lowers drug cravings. Importantly, activating the humoral immune stress response effectively reduces depression levels, lessens drug dependence, diminishes craving and negative reinforcement, and boosts drug control capability. Collectively, these effects positively contribute to increasing abstinence success rates and reducing or delaying relapse [35]. Jia Dongming's research shows that compared to conventional Eight‐Section Brocade, the LC‐modified version more effectively reduces body fat and improves lipid metabolism while maintaining equivalent safety. Additionally, based on Wang Kun's research, mindfulness‐integrated aerobic exercise demonstrates superior efficacy in reducing cravings compared to standard aerobic exercise alone. This enhanced effect may be mediated through improved sleep quality [30].

Systematic analysis reveals that different types of exercise interventions yield distinct benefits in addiction recovery: aerobic exercise primarily enhances physical function and psychological state; resistance training improves muscle strength, mental status and addiction‐related outcomes; traditional mind–body exercises focus on psychosomatic regulation and self‐control; and team sports bolster social skills, competitiveness, self‐discipline and rehabilitation efficacy. Combined exercise modalities demonstrate superior comprehensive effects on both physiological and psychological aspects. In comparative studies of single interventions, Zhao Qi found that dance therapy is more effective than cycling for rehabilitating methamphetamine‐dependent women [12]. Yao Hui's research indicates that yoga outperforms both meditation and physical training in improving outcomes, potentially due to increased kynurenic acid (KA) levels and reduced anxiety—key mediators in lowering relapse risk through exercise interventions [41]. Comparative analysis of exercise intervention efficacy can drive theoretical innovation and provide foundational support for subsequent research. Current studies on exercise interventions for addiction rehabilitation often suffer from limitations, including small sample sizes, homogeneous intervention protocols and insufficient long‐term follow‐up. Systematically comparing intervention effects directly addresses these limitations, thereby establishing new paradigms for substance rehabilitation research and informing methodological approaches for other addictive behaviours and chronic disease interventions. This generates comprehensive, robust evidence to advance the field toward precision‐focused scientific development, warranting expanded and deepened investigation. Comparative studies reveal that combined exercise modalities demonstrate broader therapeutic effects and superior comprehensive outcomes for addiction recovery compared to single‐mode interventions. Integrated approaches (e.g., pairing aerobic, resistance and traditional sports with mindfulness or group therapy) effectively address limitations of unitary interventions by filling critical gaps in multifactorial interaction research. This paradigm better elucidates neurobiological‐psychosocial synergies, enabling tailored programs based on addiction type, disease progression and physical/psychological profiles. Such combinatorial strategies—aligning with scholarly priorities as noted by Li Songyang's proposal to examine aerobic‐resistance‐pharmacotherapy‐psychotherapy integration for methamphetamine rehabilitation—are emerging as a research frontier for optimizing personalized intervention frameworks [34]. Wang Kun emphasizes that future clinical research should integrate mindfulness training with aerobic exercise while rigorously investigating causal relationships among variables. This combined approach may constitute an effective therapeutic strategy for facilitating abstinence recovery in individuals with MAUD [38]. Yao Hui notes that an integrated approach combining exercise with meditation may be more conducive to enhancing abstinence efficacy [41].

In conclusion, it is still highly necessary to conduct more global clinical randomized trials to facilitate early identification, proper management, and improve withdrawal treatment as well as the physical and psychological recovery of individuals with addiction [74, 75].

## 8 Conclusions

A synthesis of 39 core studies shows that diverse exercise interventions are effective across various addiction populations. The neurobiological basis of drug addiction includes dysregulation of dopaminergic systems, altered neuroplasticity and epigenetic regulation. Exercise counteracts these mechanisms by activating endogenous reward pathways and remodelling neural circuits, thereby significantly enhancing physiological functions, cognitive‐emotional regulation and sociocommunicative functions. Different exercise modalities offer specialized benefits: aerobic exercise focuses on psychophysiological recovery, resistance training enhances neuroadaptability, traditional mind–body practices strengthen self‐regulation capacities and ball sports facilitate social competency. Multimodal interventions, which synergistically target multiple pathways, achieve comprehensive abstinence outcomes. Future research must comparatively analyse different exercise modalities, optimize combinatorial protocols, and integrate biomedical with psychosocial mechanisms to refine rehabilitation efficacy.

## 9 Limitations and Prospects of the Study

Different exercise interventions all have positive effects on addicts. Drug addiction involves specific neurobiological mechanisms. Exercise interventions can improve multiple aspects of addicts' functioning. In the future, it is necessary to deepen research on the comparison between different exercise modalities and on combined intervention approaches and refine drug rehabilitation programs to enhance rehabilitation efficacy.

### 9.1 Limitations of the Study

The study's limitations include the following: (1) sample bias due to sex‐specific grouping of participants within compulsory rehabilitation centres, with no cross‐gender analysis conducted; (2) absence of standardized rehabilitation‐specific metrics, as current evaluation parameters predominantly utilize norms established for healthy populations; and (3) insufficient comparison of different exercise modalities and intensities, hindering the identification of optimal intervention parameters.

### 9.2 Research Prospects

Future research directions should focus on the following: (1) investigating gender‐specific responses and dose‐effect mechanisms of exercise interventions in addiction rehabilitation; (2) validating relapse reduction through long‐term follow‐ups by integrating public security surveillance data to track postdischarge outcomes of psychological and exercise therapies; (3) refining methodologies via advanced neuroimaging techniques (fNIRS/EEG) to decode neural mechanisms; (4) expanding evaluation metrics to incorporate psychosocial support and emotional factors and (5) leveraging China's compulsory rehabilitation datasets while enhancing cross‐national comparative studies.

## Author Contributions

Study concept and design: Qinghua He and Li Zhu. Analysis and interpretation of data: Zhaosong Wang and Hao Wang. Drafting of the manuscript: Zhaosong Wang and Hao Wang. Critical revision of the manuscript for important intellectual content: Yiyi Jiang and Xin Wang. Statistical analysis: Fuxuan Luo and Chaoyi Zhu. Study supervision: Changlong Zhan. All the authors have agreed to be responsible for ensuring the accuracy of the presented data.

## Funding

The authors received no specific funding for this work.

## Data Availability

The data that support the findings of this study are available on request from the corresponding author. The data are not publicly available due to privacy or ethical restrictions.
